# A large expert-curated cryo-EM image dataset for machine learning protein particle picking

**DOI:** 10.1038/s41597-023-02280-2

**Published:** 2023-06-22

**Authors:** Ashwin Dhakal, Rajan Gyawali, Liguo Wang, Jianlin Cheng

**Affiliations:** 1 Department of Electrical Engineering and Computer Science, NextGen Precision Health, University of Missouri, Columbia, MO 65211 USA; 2 Laboratory for BioMolecular Structure (LBMS), Brookhaven National Laboratory, Upton, NY 11973 USA

**Keywords:** Data publication and archiving, Cryoelectron microscopy

## Abstract

Cryo-electron microscopy (cryo-EM) is a powerful technique for determining the structures of biological macromolecular complexes. Picking single-protein particles from cryo-EM micrographs is a crucial step in reconstructing protein structures. However, the widely used template-based particle picking process is labor-intensive and time-consuming. Though machine learning and artificial intelligence (AI) based particle picking can potentially automate the process, its development is hindered by lack of large, high-quality labelled training data. To address this bottleneck, we present CryoPPP, a large, diverse, expert-curated cryo-EM image dataset for protein particle picking and analysis. It consists of labelled cryo-EM micrographs (images) of 34 representative protein datasets selected from the Electron Microscopy Public Image Archive (EMPIAR). The dataset is 2.6 terabytes and includes 9,893 high-resolution micrographs with labelled protein particle coordinates. The labelling process was rigorously validated through 2D particle class validation and 3D density map validation with the gold standard. The dataset is expected to greatly facilitate the development of both AI and classical methods for automated cryo-EM protein particle picking.

## Background & Summary

Cryo-electron microscopy (cryo-EM) is an experimental technique that captures 2D images of biological molecules and assemblies (protein particles, virus, etc.) at cryogenic temperature using ‘direct’ electron-detection camera technology^1^. With the advent of cryo-EM, there has been a boom in structural discoveries relating to biomolecules, particularly large protein complexes and assemblies. These 3D structures of proteins^2^ are important for understanding their biological functions^3^ and their interactions with ligands^4,5^, which can aid both basic biological research and structure-based drug discovery^4,6^. A key step of constructing protein structures form cryo-EM data is to pick protein particles in cryo-EM images (micrographs). Before diving into recent developments in protein particle picking and the bottleneck it faces, it is important to understand the physics and chemistry behind the grid preparation and micrograph image acquisition in cryo-EM experiments.

## Cryo-EM grid preparation and image acquisition

**Fig. 1:**
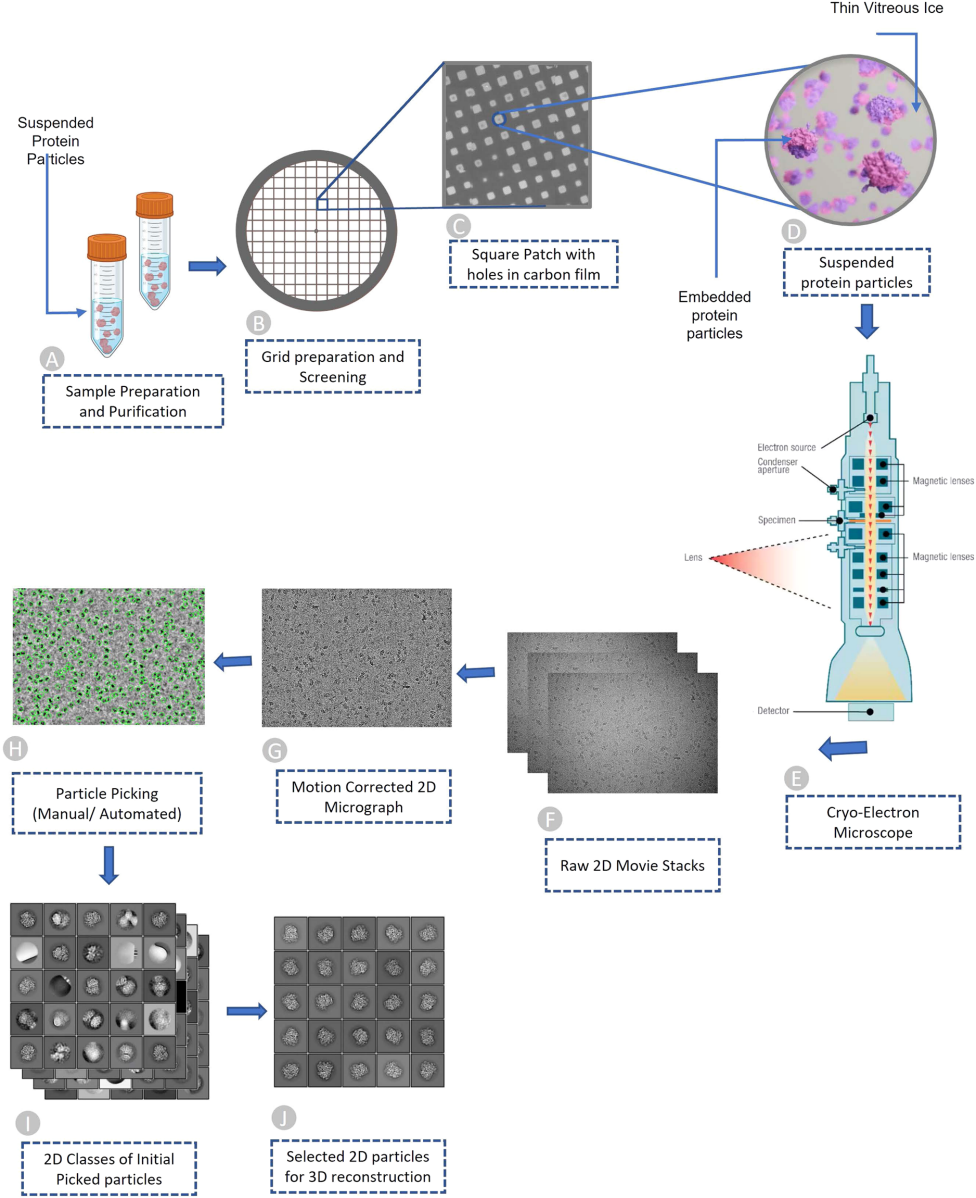
Overview of Cryo-EM pipeline, from sample preparation to particle recognition. (**A**) Aqueous sample preparation that contains variably dispersed heterogenous structure. (**B**) Cryo-EM grid containing holes that are filled with dispersed protein particles. (**C**) Magnified image of square patch illustrating microscopic holes in carbon. (**D**) Zoomed-in view of single hole containing suspended protein particles in thin layer of vitreous ice. (**E**) Cryo-Electron microscope used to facilitate high quality image generation. (**F**) Stack of 2D movie frames generated from microscope, called micrographs. (**G**) Motion corrected 2D micrograph images. (**H**) Particle picking using manual intervention or automatic procedures (green circles represent picked particles). (**I**) Initial 2D classes that contain quality protein particles along with junks and aggregates. (**J**) Best quality protein particles identified through computational analysis and visual inspection for 3D protein structure reconstruction.

The process of acquiring the two-dimensional projections of biomolecular samples (e.g., protein particles) can be summarized in four brief steps: (1) sample purification, (2) cryo-EM grid preparation, (3) grid screening and evaluation, and (4) image capturing. Once the sample is purified according to the standard protocols^7^; the next step of the single-particle procedure is to prepare the cryo-EM specimen. The grid preparation process, also known as vitrification, is straightforward. An aqueous sample is applied to a grid, which is then made thin. Eventually, the grid is plunged frozen at a time scale that inhibits the crystalline ice formation. Additionally, the particles must be evenly distributed across the grid in a wide range of orientations. It is very difficult to achieve a perfect cryo-EM grid because particles may choose to adhere to the carbon layer instead of being partitioned into holes. They may also adopt preferred orientations within the vitrified ice layer, which reduces the number of unique views^8^. The grid is ready for analysis once the cryo-EM sample is successfully inserted into the electron microscope^9^. Images are routinely captured during the screening phase at various magnifications to check for ice and particle quality. After the grids are optimized and ready for cryo-EM data collection, they are taken to a cryo-EM facility where qualified professionals load specimens into the microscope. To enable the best high-quality image capturing, experts adjust several parameters such as magnification, defocus range, electron exposure, and hole targeting techniques (see Fig. 1A–F illustrating the process of preparing cryo-EM samples and acquiring cryo-EM images). More details regarding cryo-EM sample preparation and image acquisition can be found in these studies^7,10^.

## Cryo-EM micrographs and single particle analysis

When the electron beam passes through a thin vitrified sample, it creates 2D image projections (see Fig. 1 for a visual illustration) of the samples (e.g., protein particles). The projections of the particles in various orientations are stored in different image formats (MRC, TIFF, TBZ, EER, PNG, etc.) which are called micrographs. Once the micrographs are obtained, the objective is to locate individual protein particles in each micrograph while avoiding crystalline ice contamination, malformed particles and grayscale background regions. In other words, the input for the particle picking problem is a micrograph, while the desired output is the coordinates of every protein particle in that micrograph (refer to Fig. 1 for the entire pipeline). Accurate detection of particles is necessary, as the presence of false positive particles can complicate subsequent processing, and eventually cause the 3D reconstruction process to fail entirely. The picking task is challenging due to several factors, including high noise levels caused by ice and contamination, low contrast of particle images, particles with heterogenous conformations, and unpredictability in an individual particle’s appearance caused by variation in orientation. Once the particles are extracted from the micrographs, single particle analysis is performed to reconstruct the 3D density map and protein structure.

## Advances and challenges in single protein particle picking

Several research initiatives were carried out worldwide to improve hardware^11–13^ and software^14–17^ to streamline and automate the cryo-EM data collection and processing steps for the determination of 3D structures^18^. The recent technological advances in sample preparation, instrumentation, and computation methodologies have enabled the cryo-EM technology to solve many protein structures at better than 3 A resolution. To obtain a high-resolution protein structure, selecting enough high-quality protein particles in cryo-EM images is critical. However, protein particle picking is still largely a computationally expensive and time-consuming process. One challenge facing cryo-EM data analysis is to develop automated particle picking techniques to circumvent manual intervention. To tackle the problem, numerous automatic and semi-automatic particle-picking procedures have been developed.

A common technique for particle picking, known as template matching, uses user-predefined particles as templates for identifying particles in micrographs through image matching. However, because of varied ice contamination, carbon areas, overlapping particles, and other issues, the template matching often selects invalid particles (e.g., false positives). So subsequent manual particle selection is necessary.

To deal with the issue, artificial intelligence (AI) and machine learning-based approaches have been proposed, which can be less sensitive to impurities and more suitable for large-scale data processing and therefore hold the potential of fully automating the particle picking process. XMIPP^19^, APPLE picker^20^, DeepPicker^21^, DeepEM^22^, FastParticle Picker^23^, crYOLO^24^, PIXER^25^, PARSED^26^, WARP^27^, Topaz^28^, AutoCryoPicker^29^, and DeepCryoPicker^30^, can be taken as good examples of such efforts.

The datasets used to train and test machine learning particle picking methods were curated from EMPIAR^31^. It contains almost all the publicly available raw cryo-EM micrographs. It is a public repository containing 1,159 entries/datasets (2.39 PB) as of Jan 29, 2023. It includes not just cryo-EM images of proteins, but also Soft X-ray Tomography (SXT), cryo-ET and many other microscopic projections of other biological samples. Only some cryo-EM images of a small number of datasets in EMPIAR contain particles manually labelled by the original authors of the data. Therefore, most existing machine learning methods for particle picking were trained and tested on only a few manually labeled datasets of a few proteins like Apoferritin and Keyhole Limpet Hemocyanin (KLH). The methods trained on the limited amount of particle data of one or a few proteins cannot generalize well to pick particles of various shapes in the cryo-EM micrographs of many diverse proteins in the real world. Therefore, even though machine learning particle picking is a promising direction, no machine learning method has been able to replace the labor-intensive template-based particle picking in practice. Therefore, the lack of manually labelled particle image data of diverse protein types is hindering the development of machine learning and AI methods to automate protein particle picking.

Creating a high-quality manually labelled single-protein particle dataset of a large, diverse set of representative proteins to facilitate machine learning is a challenging task. Single-particle cryo-EM images suffer from high background noise and low contrast due to the limited electron dose to minimize the radiation damage to the biomolecules of interest during imaging, which makes particle picking difficult even for humans. Low signal-to-noise ratio (SNR) of the micrographs, presence of contaminants, contrast differences owing to varying ice thickness, background noise fluctuation, and lack of well-segregated particles further increases the difficulty in particle identification^32^. This is one reason there is still a lack of large manually curated protein particle datasets in the field.

A common problem of the particle picking algorithms trained on a small amount of particle data of a few proteins is that they cannot distinguish ‘good’ and ‘bad’ particles well, including overlapped particles, local aggregates, ice contamination and carbon-rich areas^33^. For instance, the methods: DRPnet^34^, TransPicker^35^, CASSPER^36^, and McSweeney *et al.’s* method^37^ that made significant contributions to the particle selection problem suffered the two similar problems. Firstly, there is not a sufficient and diversified dataset to train them. Secondly, there are not enough manually curated data to test them. The similar problems happened to other supervised and unsupervised machine learning methods, such as an unsupervised clustering approach^38^, AutoCryoPicker^29^, DeepCryoPicker^30^, APPLE picker^20^, Mallick *et al.’s* method^39^, gEMpicker^40^, Langlois *et al.’s* method^33^, DeepPicker^21^, DeepEM^22^, Xiao *et al.’s* method^23^, APPLE picker^20^, Warp^27^, SPHIRE-crYOLO^41^, and HydraPicker^42^ all encountered similar problems. They usually perform well on the small, standard datasets used to train and test them (e.g., Apoferritin and KLH), but may not generalize well to non-ideal, realistic datasets containing protein particles of irregular and complex shapes, which are generated daily by the cryo-EM facility around the world.

To address this key bottleneck hindering the development of machine learning and AI methods for automated cryo-EM protein particle picking, we created a large dataset of cryo-EM micrographs, named CryoPPP^43^, which includes manually labelled protein particles. The micrographs are associated with 34 representative proteins of diverse sequences and structures that cover a much larger protein particle space than the existing datasets of a few proteins such as Apoferritin and KLH.

In this study, we ensured the inclusivity of diverse protein particles by considering various protein types, shape patterns, sizes (ranging from 77.14 kDa to 2198.78 kDa), source organisms, and different variations of the micrographs. Supplementary Table S3 provides the additional information on the different protein types that were selected to label particles. Additionally, we incorporated cryo-EM images with varying defocus values associated with each EMPIAR ID to include a diverse set of micrographs. The box-whisker plot in Supplementary Fig. S1 shows the diversity of the defocus values in each EMPIAR ID.

**Fig. 2:**
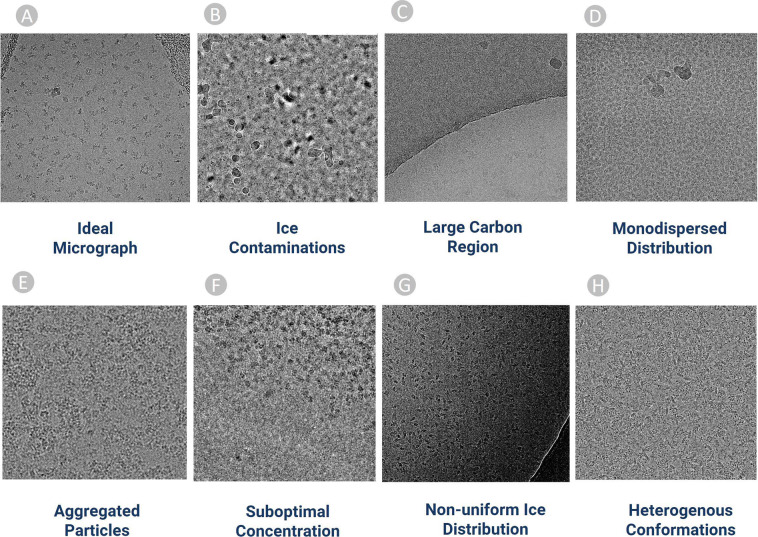
Examples of diverse cryo-EM micrographs in CryoPPP used for particle labelling. (**A**) Ideal micrographs (EMPIAR-10590) containing protein particles with sufficient contrast that can be easily identified by naked eye. (**B**) Micrographs containing an abundance of ice patches and contaminations (EMPIAR-10532). Crystalline ice is evident by the cluster of light and dark patches making it difficult to distinguish true protein particles from false positives. (**C**) Micrograph containing large carbon areas (EMPIAR-10005). (**D**) Micrographs containing a monodisperse distribution of protein particles (EMPIAR-10852). (**E**) Large Cluster of clumped protein particles making it difficult to recognize and pick particles (EMPIAR-10387). (**F**) Micrographs (EMPIAR-11057) containing protein particles overcrowded at upper half region. (**G**) Difference of ice thickness resulting in the top left part to appear brighter and the bottom right part darker. A crack within the vitrified hole in the lower right part of micrograph causing blurring effect (EMPIAR-10017). (**H**) Micrograph (EMPIAR-10093) containing heterogeneous top, side, and inclined views of protein particles.

Machine learning methods yield best results when being trained on a diverse set of representative data. Hence, we included micrographs containing particles with different physical features and complexities, as shown in Fig. 2. Figure 2A–D depict the most ideal cases of particle picking in which the protein particles are visible to naked eyes. If machine learning methods are only trained solely on these micrographs, they perform relatively well in the simple cases but poorly on more challenging micrographs that have low signal-to-noise ratio. Thus, we selected difficult micrographs that contain both particles and lots of ice patches, contaminations (Fig. 2B) and carbon regions (Fig. 2C).

In addition to the aforementioned variations in the micrographs, CryoPPP also comprises micrographs containing mono-dispersed (Fig. 2D) particles characterized by particles of uniform size and micrographs with heterogenous confirmations that have varying top, side and inclined views of particles. Other micrographs with sub-optimal concentration of particles, clusters of proteins, and non-uniform ice distributions were included. The detailed features of the 34 diverse sets of cryoEM micrographs in CryoPPP are described in Supplementary Table S3.

The quality of the manually labeled particles of selected proteins was eventually validated rigorously. This validation was done against particles originally labelled by the authors who generated the cryo-EM data (called gold standard here) by both 2D particle class validation and 3D cryo-EM density map validation. The quality of our manual annotation is on par with the annotations provided by the experts who created the data in the first place, which confirms our manual particle labelling process is effective. Therefore, we believe CryoPPP is a valuable resource for training and testing machine learning and AI methods for automated protein particle picking.

## Methods

**Fig. 3:**
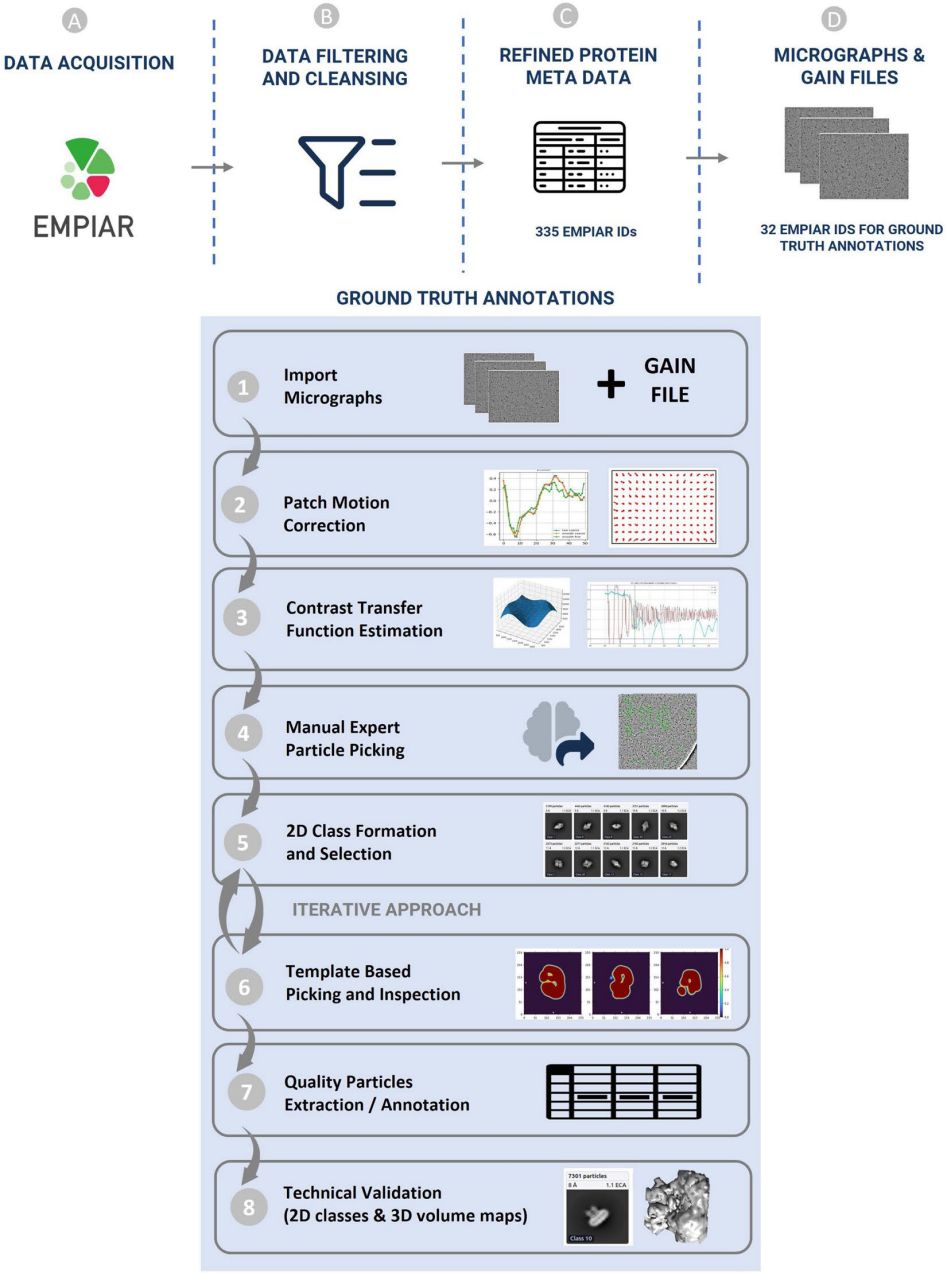
Graphical illustration of the overall methodology of creating CryoPPP dataset. (**A**–**D**) represent the steps for data acquisition and protein metadata preparation. (1–8) represent subsequent steps for the ground truth annotation and validation of picked protein particles. The iterative approach between step (5) and (6) is carried out to achieve the high-quality picking of particles.

CryoPPP was created through a series of steps as shown in Fig. 3. We first crawled the data from the EMPIAR website using python API and FTP scripts fetched through Bash scripting. We filtered out microscopic images of various non-single-protein particles (e.g., bacteria, filaments, RNA, protein fibril, virus-like particles) and retained only high-resolution micrographs acquired by cryo-EM technique for manual particle labeling.

After importing the micrographs with all the physiochemical parameters gathered from the corresponding published literature, we performed motion correction and Contrast Transfer Function (CTF) estimation for them. After preparing the micrographs, two human experts conducted an initial manual particle selection process separately, using low pass filter values and appropriate particle diameter settings, on approximately 20 out of about 300 micrographs per selected protein (EMPIAR ID).

The expert-picked particles were cross-validated and then went through 2D particle classification. The best particles based on resolution, particle count, and visually appealing and sensible 2D classes were selected and further used for template-based particle picking and further human inspection. After iterating the 2D classes from template-based picking and human inspection, we ultimately obtained the final set of highly confident protein particles as ground truth and exported them in the files in star, csv and mrc formats. The first two files (.star and.csv) contain the coordinates of the protein particles and the latter (.mrc) store particle stacks. The process of creating CryoPPP in Fig. 3 is described in the following sections in detail.

### EMPIAR metadata collection and filtering

The process of preparing the dataset began with collecting metadata about cryo-EM image datasets in EMPIAR. Data collection scripts that use python API and FTP protocols were used to automatically download the metadata from the EMPIAR web portal^31^. The metadata includes EMPIAR ID of each cryo-EM dataset of a protein, the corresponding Electron Microscopy Data Bank (EMDB) ID, Protein Data Bank (PDB) ID, size of dataset, resolution, total number of micrographs, image size/type, pixel spacing, micrograph file extension, gain/motion correction file extension, FTP path for micrograph/gain files, Globus path for micrograph/gain files, and publication information.

**Fig. 4:**
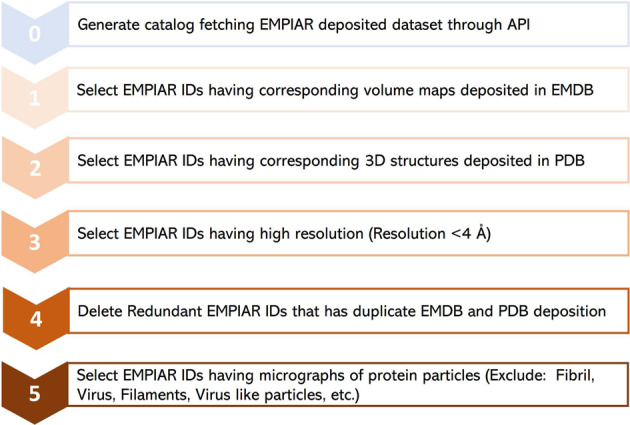
The step-by-step procedure for collecting and selecting Cryo-EM protein datasets from EMPIAR database. 335 unique EMPIAR datasets (IDs) of 335 proteins were selected at the end.

Following the metadata collection, the individual cryo-EM datasets in the collection were filtered as depicted in Fig. 4 (**Steps 1–5**). First, we only chose EMPIAR IDs (datasets) that have their volume maps deposited in EMDB. From the chosen EMPIAR datasets, we only selected ones that had corresponding protein structures in the Protein Data Bank (PDB).

To ensure high data quality, we then retained only the EMPIAR datasets whose resolution was better than 4 Angstrom (Å). We observed that there were some redundant EMPIAR datasets (e.g., EMPIAR ID: 10709 & 10707, EMPIAR ID: 10899 & 10897) that correspond to the same biomolecule with the same PDB and EMDB IDs. Hence, we eliminated those duplicate entries. After removing duplicate records, we selected only EMPIAR datasets that contained micrographs of protein particles, excluding other biological samples such as viruses. This filtering step required some literature study of individual EMPIAR datasets. The motion correction and gain correction files for the selected datasets were extracted from the EMPIAR if required. The final list of meta data includes 335 EMPIAR entries, 34 out of which were used for manual labelling. Refer to the *EMPIAR_metadata_335.xlsx* file in CryoPPP for further information about the list of 335 datasets of 355 proteins.

### Manual particle labeling

Manually picking particles in cryo-EM micrographs through the GUI interfaces of cryo-EM analysis tools such as CryoSPARC^16^, EMAN2^14^ and RELION^15^ is very time consuming. We carefully imported large micrographs, carried out the motion correction, and estimated CTF, especially in the case when flipping and rotating gain files are required to match the input array shape of the micrograph and gain file. Furthermore, it takes a lot of disk space to store the labelled particle data together with the corresponding micrographs and particle stack files. Therefore, we chose 34 representative EMPIAR datasets out of 335 entries selected in the previous section for manual particle labelling to create the CryoPPP dataset, considering diverse particle size/shapes, density distribution, noise level, and ice and carbon areas. Moreover, proteins from a wide range of categories, such as: metal binding, transport, membrane, nuclear, signaling, and viral proteins were selected. See supplementary Tables S1, S3 for more details about the 34 proteins (cryo-EM datasets). Most of the pre-processing, manual particle labelling, real-time quality assessment, and decision-making workflows were performed using CryoSPARC v4.1.1^16^, EMAN2^14^, and RELION 4.0^15^.

CryoPPP includes a total of 9,893 micrographs (~300 Cryo-EM images per selected EMPIAR dataset). We labelled ~300 micrographs per EMPIAR data because using all the micrographs in each dataset would result in 32.993 TB of data, making it too large to store, transfer, and use for most machine learning tasks.

There is no rule of thumb to determine the number of particles required for training machine learning methods to achieve optimal performance. It varies from protein to protein. It is also worth noting that the resolution of 3D density map is not solely dependent on the number of picked 2D particles, but also on the inclusion of a diverse range of particle orientations, which can significantly improve the resolution. CryoPPP contains 2.7 million particles of 34 different proteins, which should be adequate for machine learning algorithms to learn relevant particle features. Typically, we utilized approximately 300 micrographs per protein to pick particles. However, if adequate particle views were not extracted from the first 300 micrographs, a larger number of micrographs were employed. The different orientations of protein particles were assessed during the 2D classification and manual inspection stages which are elaborated in the subsequent sections.

#### Importing movies

This is the crucial first step of particle labeling. For each EMPIAR dataset, we import two inputs: micrographs and gain reference (motion correction files). We analyzed the description of the EM data acquisition and grid preparation for each dataset in order to collect the important information such as raw pixel size (Å), acceleration voltage (kV), spherical aberration (mm), and total exposure dose (e/Å^2^) for the micrographs in the dataset.

Furthermore, we obtained gain reference for micrographs if their motion was not corrected before. We used *e2proc2d*, a generic 2-D image processing program in EMAN2^14^, to convert different formats of motion correction file (e.g.,.dm4,.tiff,.dat, etc.) to.mrc file since CryoSPARC accepts only.mrc extension. Then, based on the microscope camera settings and how the data was acquired during the imaging process, we applied geometrical transformations (flip gain reference and defect file left-to-right/top-to-bottom (in x/y axis) or rotate gain reference clockwise/anti-clockwise by certain degrees) relative to the image data. Supplementary Table S1 contains the details of input parameters for each EMPIAR ID. After importing movies and motion correction files, we proceeded to the job inspection panel of CryoSPARC to ensure that all input settings and loaded micrographs were correct.

#### Patch motion correction

When specimens are exposed to an electron beam, the mobility of sample molecules (protein particles) during data acquisition can affect the overall quality of electron micrographs and lower the final resolution^44^. Hence, it is necessary to correct the movement of particles (referred to as ‘beam-induced motion’).

The causes behind this motion can be categorized into two types: (1) Motion from Microscope: It is caused by stage drift and usually occurs in microscope due to little amount of vibration left over after the stage has been aligned to a new position^45^. It moves the sample relative to the beam and optical axis. This motion is quite jagged in time, with sharp accelerations or twitches, but is consistent. The entire image will move in the same direction over time. (2) Motion from sample deformation: This motion is caused by the energy deposited into the ice by the beam, or energy already trapped in it, due to strained forces locked in during freezing. It is eventually released during the image capturing process. As the electrons pass through the samples, the energy from the beam and the temperature change causes the ice to physically deform and bend. That deformation is often smoother over time, but it can be highly anisotropic in space. In this case, various parts of the same image can move in different directions at the same time.

**Fig. 5:**
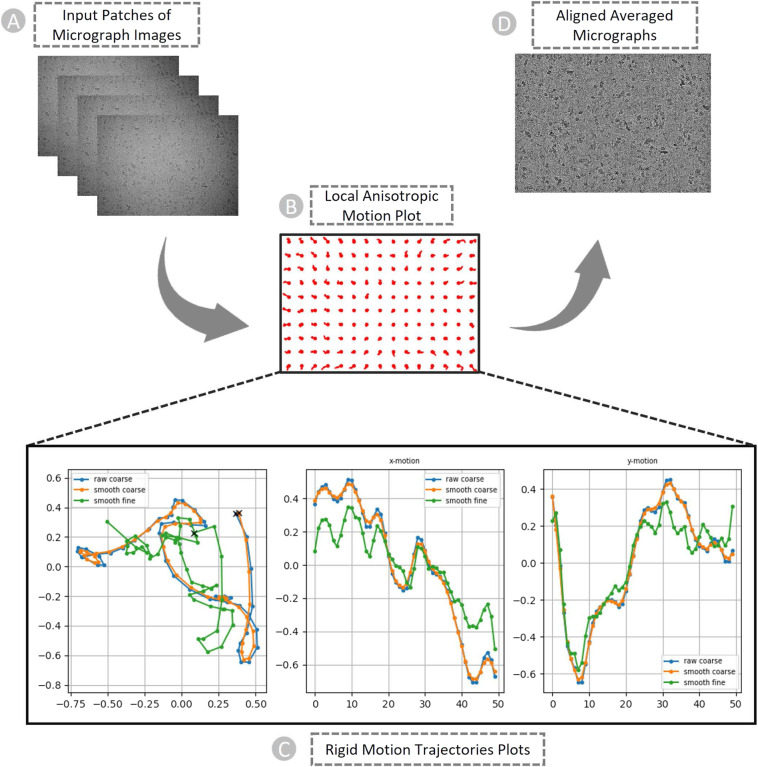
The patch-based local and global motion correction pipeline for EMPIAR ID 10737 (E. coli cytochrome bo3 in MSP Nanodiscs). (**A**) Full frames of micrographs as input. (**B**) Anisotropic deformation. (**C**) Rigid motion trajectories plots. Blue: original trajectory, Radish: trajectory with small smoothing penalty, Green: trajectory with fine smoothing. Left: Overall motion trajectory over X and Y motion. Center: X-motion plot over time. Right: Y-motion plot over time. (**D**) Non-dose weighted aligned averaged micrographs with the highest amount of signal and least amount of motion blur as output.

Both motions must be estimated and corrected to obtain high-resolution reconstructions from the data. In the patch-based motion correction step, we corrected both global motion (stage drift) and local motion (beam-induced anisotropic sample deformation) for the micrographs (as shown in Fig. 5A using CryoSPARC. In the anisotropic deformation plot in Fig. 5B, each red circle indicates the center of a single “patch” of the image, and the curves emerging from each circle show the motion of that portion of the sample. We can observe the correlation between the motion of adjacent patches. They move somewhat similarly to one another. To prevent the fit from being distorted by random noise in the micrograph, the patch motion correction algorithm imposes smoothness constraints on the motion.

Figure 5C are the examples of plots generated by patch motion correction that depict the computed trajectories. The set of plots shows overall motion correction (an actual trajectory plot, followed by X-motion plot and Y-motion plots over time). In the overall motion trajectory over X and Y motion (Fig. 5C, **Left**), each dot represents the sample’s position from frame to frame. Here, the x and y axes represent the units of pixels in the raw data’s pixel size. The sample begins at point (**X**), moves downward, makes a curve and again changes direction toward the left-top, and then continues to descend to the left. We apply this trajectory to the input data by shifting each image in reverse of what the motion trajectory suggests and finally averaging images together. In other words, we track a sample’s motion during the exposure to undo it.

While curating CryoPPP, we noticed several factors (protein size, charge, and ice thickness) potentially influencing the scale of local motion in Cryo-EM micrographs. Larger proteins tend to exhibit slower local motion than smaller ones, due to their increased mass and higher structural complexity. The charge distribution on a protein surface can also affect the scale of local motion, with highly charged proteins exhibiting more restricted motion due to the electrostatic interactions with the surrounding ice, while relatively neutral proteins may be more mobile. Additionally, we observed that the thickness of the ice layer surrounding a protein can influence local motion; thicker ice layers can provide more structural stability but may introduce more noise and distortion in the micrograph.

#### Patch-based CTF estimation

The contrast of images captured in the electron microscope is affected by imaging defocus and lens aberrations, which are adjusted by microscope operators to enhance the contrast. The relationship between lens aberrations and the contrast in the image is defined by the CTF. It explains how information is transferred as a function of spatial frequency.

It is important to estimate CTF, which is then corrected during 2D particle classification and 3D reconstruction steps. Otherwise, the feasible reconstruction will have extremely low resolution. A full treatment of the effects of the CTF usually proceeds in two stages: CTF estimation and CTF correction. In CryoSPARC used in this work, the CTF model is given by the Eq. 1.

1 $${C}{T}{F}=-{c}{o}{s}\left({\pi }\Delta {z}{{\lambda }}_{{e}}{f}^{2}-\frac{{\pi }}{2}{C}_{{s}}{{\lambda }}_{{e}}^{3}{f}^{4}+{\Phi }\right)$$

where Δ*z* is defocus, *λ*_*e*_ is the wavelength of the incident electrons, *C*_*s*_ *is* spherical aberration, and *f* is spatial frequency. *Φ* represents a phase shift factor.

Most cryo-EM samples are not ‘flat’. Before a sample is frozen, particles tend to concentrate around the air-water interfaces, and the ice surface itself is usually not flat^46,47^. Because defocus has an impact on the CTF, distinct particles can have various defoci and hence various CTFs within a single image. To address this problem, CryoSPARC offers a patch-based CTF estimator that analyzes numerous regions of a micrograph to calculate a “defocus landscape”.

**Fig. 6:**
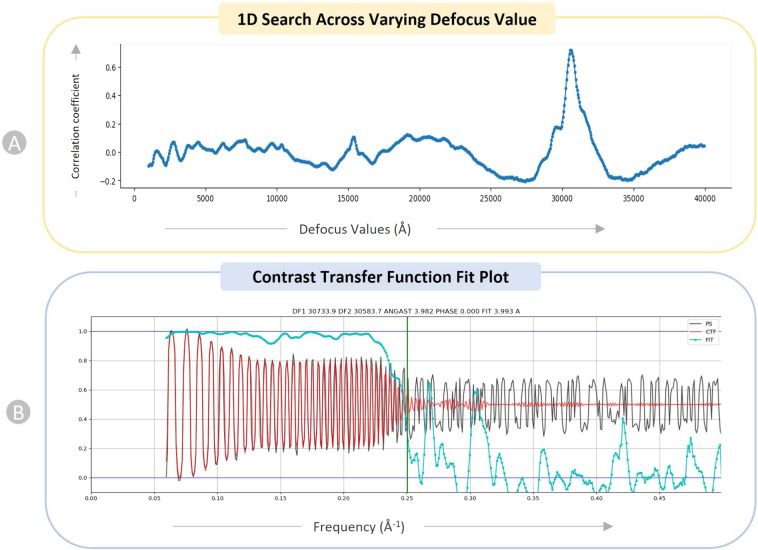
Diagnostic plots of CTF for EMPAIR 10737 (E. coli cytochrome bo3 in MSP Nanodiscs). (**A**) 1D search over varying defocus values (underfocus). (**B**) CTF fit plot. X-axis displays frequency, in units in inverse angstroms (Å^−1^) and Y-axis shows correlation metric between power spectrum (PS) and CTF value. Black: observed experimental power spectrum. Red: calculated CTF. Cyan: cross-correlation (fit).

We performed a 1D search over defocus for every micrograph. Figure 6A depicts the 1D search for a particular micrograph of EMPIAR ID 10737^48^. This plot helps identify a particular defocus value that stands out among a variety of other defocus values (x-axis). Patch CTF creates a plot showing how closely the input micrographs’ observed power spectrum and the calculated CTF match. The CTF fit plot in Fig. 6B shows that the computed CTF matches the observed power spectrum up to a resolution of 3. 993 Å. The cross correlation between the observed spectrum and the calculated CTF is depicted by the cyan line in the plot. The vertical green line in the plot represents the frequency at which the fit deviates from CryoSPARC’s cross-correlation threshold of 0.3 for a successful fit.

We executed the patch CTF to obtain the output micrographs with data on their average defocus and the defocus landscape. When particles were extracted, this data was automatically used to assign each particle a local defocus value based on its position in the landscape.

#### Manual particle picking

After performing the motion correction and CTF estimation, we manually picked particles interactively from aligned/motion-corrected micrographs with the goal of creating particle templates for auto-picking using ‘Manual Picker‘ job in CryoSPARC. Depending on the size and shape of the protein particles, we adjusted the box size and the particle diameter. Since picking particles on raw micrographs is extremely difficult, we tweaked the ‘Contrast Intensity Override’ while viewing micrographs in order to obtain the best distinctive view for picking particles.

**Fig. 7:**
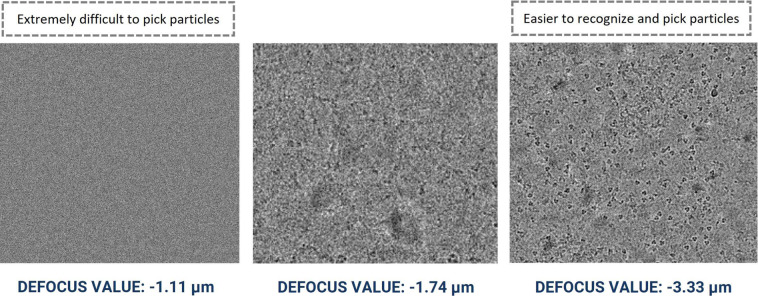
Cryo-EM micrograph images of EMPIAR ID 10532 (Influenza Hemagglutinin) with different defocus values. Micrographs with smaller defocus values make particle picking difficult and vice-versa.

It is particularly challenging to manually pick particles from micrographs with smaller defocus levels. Figure 7 illustrates the visualization of micrographs in the same dataset with different defocus levels for EMPAIR 10532^48^. It is worth pointing out that the task of particle picking becomes significantly more challenging for AI methods when the micrographs have a low signal-to-noise ratio, contain numerous ice-patches, carbon areas, aggregated particles, and non-uniform ice distribution, as illustrated in Fig. 2B,C,E,G). Hence, to generate comprehensive templates, we manually picked particles from diverse micrographs and the micrographs with wide defocus range (see supplementary Fig. S1) and CTF fit values.

As manual picking was very time intensive, we selected a subset of micrographs (around 20 micrographs of each EMPIAR dataset) for manually picking initial particles for the subsequent template-based particle picking.

To ensure data reproducibility, the intermediate star files of manually expert-picked particles are deposited in CryoPPP and can be found under the *ground-truth* subdirectory. Additional information regarding the total number of manually picked particles, number of micrographs considered for manual picking, particle diameter, angular sampling, and minimum separation distance of particles are provided in the Supplementary Table S2.

#### Forming and selecting best 2D particle classes

The manually picked particles went through the 2D classification step. This step helped to classify the picked particles into several 2D classes to facilitate stack cleaning and junk particles removal. To analyze the distribution of views within the dataset qualitatively, we specified a specific number of 2D classes. By doing this, we investigated the particle quality and removed junk particle classes, which ultimately facilitated the selection of good particle classes.

We specified the initial Classification Uncertainty Factor (ICUF) and maximum alignment resolution to align particles to the classes with 40 expectation maximization (EM) iterations. The diameter of the circular mask that was applied to the 2D classes at each iteration was controlled using the circular mask diameter in the case of crowded particles.

**Fig. 8:**
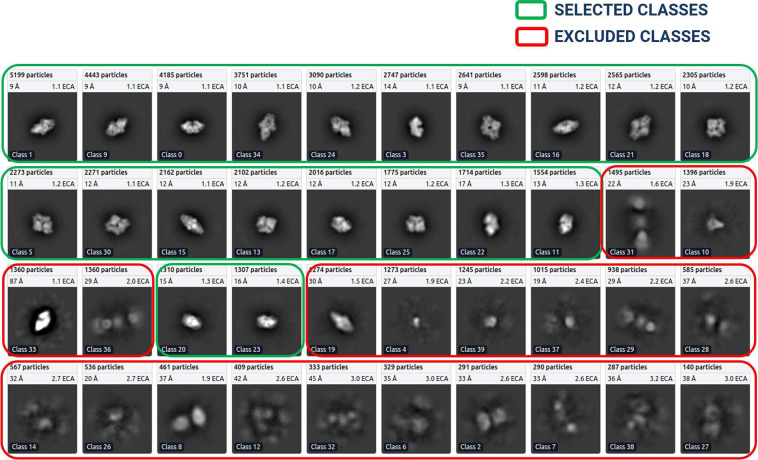
2D classes for EMPIAR ID 10017 (Beta-galactosidase), ordered ascendingly by the number of particles assigned to each class. Green: High quality particle classes selected for further template-based picking. Red: Rejected particle classes.

After the 2D classes were formed, we selected the best particle classes interactively to remove the junks. Figure 8 shows an example of 2D classification and selection of highly confident particles for EMPIAR ID 10017^49^. We used three diagnostic measures to select the 2D classes: resolution (Å) of a class, the number of particles of a class (higher, better), visual appearance of a class. Considering only the number of particles in a class is not sufficient because some classes containing a small number of particles may represent a unique view of the protein.

Refer to the Supplementary Table S2 for additional details regarding the number of 2D classes in each EMPIAR dataset, window inner and outer radii, recenter mask threshold, number of iterations to anneal sigma as well as other relevant thresholds and parameters.

#### Template based picking and manual inspection and extraction of particles

After the best particle classes were selected and exported, we used a template generated from the ‘**Forming and Selecting Best 2D**’ step to pick more particles. The process was iterative, meaning that the output of a round of ‘template-based picking and inspection’ was again utilized for ‘2D class formation’ step to form and select best 2D classes under the human inspection. This process was repeated until we acquired high resolution particles that include all possible particle projection angles.

**Fig. 9:**
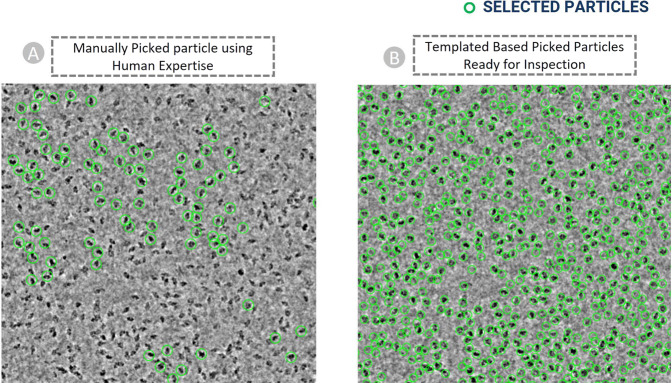
Cryo-EM micrograph image of EMPIAR ID 10017 (defocus value: −3.63 µm) used for template-based particle picking. (**A**) Micrograph with manually picked protein particles (encircled with green circle, particle diameter: 190 Å, low pass filter value: 25). (**B**) Intermediate picked protein particles with template-based picking ready for manual inspection and the adjustment of power value and NCC score.

The final templates with green boxes (as shown in Fig. 8) were used to execute auto-pick particles from micrographs. With CryoSPARC’s Template Picker, we used high resolution templates to precisely select particles that matched the geometry of the target structure. Figure 9A represents manually picked particles for EMPIAR-10017^49^ that work as templates to facilitate template-based picking that eventually results in template-based picked particles ready for human inspection as shown in Fig. 9B. We specified constraints like particle diameter in angstrom (see Supplementary Table S2 for more information) and a minimum distance between particles to generate the templates based on the SK97 sampling algorithm^34^ to remove any signals from the corners and prevent crowding. We observed that the blob-based in picking in RELION required minimum and maximum allowed diameter of the blobs, whereas defining a single value for particle’s diameter works well in CryoSPARC’s template particle picking step.

Finally, the particles obtained by the template picking went through the manual inspection step, where we examined and modified picks using various thresholds. We adjusted the lowpass filter, optimum power score range, and normalized cross-correlation (NCC) score (see Supplementary Table S2) to improve the visibility of the picks. In this step, we removed false positive particles as depicted in Fig. 12B. While efforts were made to minimize false negatives during particle picking, it is important to recognize that the complete elimination of false negatives is impossible in one round of annotation. The 2D colored histogram plots as depicted in Fig. 12 were used to scrutinize micrograph median pick scores versus defocus for extracting the coordinates of high-quality protein particles. Hence, we have strived to optimize particle picking, aiming to minimize both false positives and false negatives and thus improving the accuracy and reliability of CryoPPP. We will continue to improve and update CryoPPP throughout its life cycle, considering the input from external users.

## Data Records

The CryoPPP dataset^43^ consists of manually labelled 9,893 micrographs of 34 diverse, representative cryo-EM datasets of 34 protein complexes selected from EMPIAR. Each EMPIAR dataset identified by a unique EMPIAR ID has about ~300 cryo-EM images in which the coordinates of protein particles were labeled and cross-validated by two experts aided by software tools. The total size of CryoPPP is 2.6 TB. Additional statistical information about CryoPPP can be found in Table 1.

**Table 1:** The statistics of each EMPIAR protein dataset in CryoPPP (* Theoretical weight).

SN	EMPAIR ID	Protein Type	Size (TB)	Number of Micrographs	Image size	Particle Diameter (px)	Total Structure Weight (kDa)	Number of True Protein Particles
1	10389^56^	Metal Binding Protein	0.224	300	(3838, 3710)	313	1042.17	10870
2	10081^53^	Transport Protein	0.052	300	(3710, 3838)	154	298.57	39352
3	10289^57^	Transport Protein	0.048	300	(3710, 3838)	162	361.39	61517
4	11057^58^	Hydrolase	2.100	300	(5760, 4092)	186	149.43	45219
5	10444^59^	Membrane Protein	2.399	300	(5760, 4092)	217	295.89	58731
6	10576^60^	Nuclear Protein (DNA)	0.722	295	(7420, 7676)	265	290.21	75220
7	10816^61^	Transport Protein	1.500	300	(7676, 7420)	359	166.62	45363
8	10526^62^	Ribosome (50 S)	0.460	294	(7676, 7420)	482	1085.81	3265
9	11051^63^	Transcription/DNA/RNA	2.300	300	(3838, 3710)	214	357.31	83227
10	10760^64^	Membrane Protein	0.199	300	(3838, 3710)	106	321.69	173664
11	11183^65^	Signaling Protein	0.326	300	(5760, 4092)	159	139.36	80014
12	10671^66^	Signaling Protein	1.600	298	(5760, 4092)	133	77.14	69012
13	10291^57^	Transport Protein	0.016	300	(3710, 3838)	130	361.39	99808
14	10669^67^	Proteasome (Plant Protein)	13.899	300	(7676, 7420)	730	1681.81	19660
15	10077^68^	Ribosome (70 S)	0.774	300	(4096, 4096)	216	2198.78	31919
16	10061^69^	Hydrolase (Beta-galactosidase)	0.319	300	(7676, 7420)	471	467.06	35218
17	10028^52^	Ribosome (80 S)	1.100	300	(4096, 4096)	224	2135.89	26391
18	10096^70^	Viral Protein	1.199	300	(3838, 3710)	84	150*	231351
19	10737^48^	Membrane Protein (E-coli)	0.831	293	(5760, 4092)	179	155.83	59265
20	10387^71^	Viral Protein (DNA)	0.105	300	(3710, 3838)	213	185.87	101778
21	10532^48^	Viral Protein	0.196	300	(4096, 4096)	174	191.76	87933
22	10240^72^	Lipid Transport Protein	0.111	300	(3838, 3710)	156	171.72	85958
23	10005^73^	TRPV1 Transport protein	0.044	30	(3710, 3710)	142	272.97	5374
24	10017^49^	β -galactosidase	0.005	84	(4096, 4096)	108	450*	49391
25	10075^74^	Bacteriophage MS2	0.046	300	(4096, 4096)	233	1000*	12682
26	10184^75^	Aldolase	0.084	300	(3838, 3710)	118	150*	219849
27	10059^75^	Transport Protein (TRPV1)	0.062	295	(3838, 3710)	132	317.88	190398
28	10406^55^	Ribosome (70 S)	0.141	300	(3838, 3710)	212	632.89	24703
29	10590^51^	TRPV1 with DkTx and RTX	0.252	300	(3710, 3838)	158	1000*	62493
30	10093^76^	Membrane Protein	0.097	300	(3838, 3710)	172	779.4	56394
31	10345^54^	Signaling Protein	0.085	300	(3838, 3710)	149	244.68	15894
32	11056^77^	Transport Protein	0.164	361	(5760, 4092)	164	88.94	125908
33	10852^78^	Signaling Protein	0.227	343	(5760, 4092)	123	157.81	310291
34	10947^79^	Viral Protein	0.048	400	(4096, 4096)	240	443.92	106393

The full dataset is available at https://github.com/BioinfoMachineLearning/cryoppp. For researchers who have limited disk space, a much smaller light version of CryoPPP, called CryoPPP_Lite, can also be downloaded from the website. CryoPPP_Lite includes the micrograph files in the 8-bit JPG format and the particle ground truth files that only need 121 GB disk space in total, which is easier to store and transfer.

**Fig. 10:**
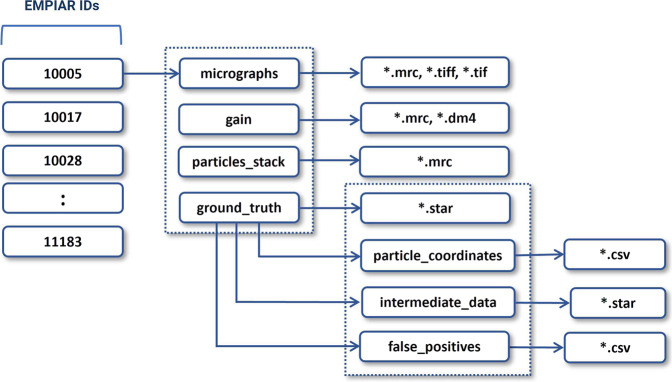
The directory structure of each expert-labelled data entry of CryoPPP. The directory contains micrographs, motion correction files, particle stacks, and ground truth labels (manually picked particles). The blocks with numbers on the left represent corresponding EMPIAR IDs.

Each data folder (named by its corresponding EMPIAR ID) includes the following information: original micrographs (either motion-corrected or not), gain motion correction file, new motion-corrected micrographs (if original micrographs are not motion-corrected), ground truth labels (manually picked particles), and particles stack. The directory structure of each data entry is illustrated in Fig. 10. The data in each directory is described as follows. It is worth noting that if the original micrographs were not motion-corrected, we applied the motion correction to them to create their motion-corrected counterparts.

### Raw micrographs

These are the two-dimensional projections of the protein particles in different orientations stored in different image formats (MRC, TIFF, EER, TIF, etc.). They can be considered as the photos taken by cryo-EM microscope. Original micrographs are from EMPIAR and can be either motion corrected or not. If an entry has a ‘*gain’* folder, it includes both raw non-motion-corrected micrographs and their motion-corrected counterparts created by us. Users are supposed to use the motion corrected micrographs as input for machine learning tasks. The scripts for the motion correction are available at CryoPPP’s GitHub website.

### Motion correction (gain files)

It contains motion correction files (if motion in original micrographs not corrected before) stored in different formats like dm4 and mrc. It is used to correct both global motion (stage drift) and local motion (beam-induced anisotropic sample deformation) that occur when specimens (protein particles) are exposed to the electron beam during imaging. Correcting the motion enables the high-resolution reconstruction from the data.

### Particle stack

Particle stack comprises of the mrc files (with names corresponding to individual micrographs’ filenames) of manually picked protein particles (ground truth labels). These are three-dimensional grids of voxels with values corresponding to electron density (i.e., a stack of 2D images). To browse and examine this file, utilize EMAN2^14^, UCSF Chimera^50^, or UCSF ChimeraX^51^.

### Ground truth labels

Ground truth data contain the star and CSV files for both all true particles (positives) and some typical false positives (e.g., ice contaminations, aggregates, and carbon edges). The positive star (and corresponding CSV) files are the ground truth position of the picked particles combined in a single file for all ~300 micrographs per EMPIAR ID. While the negative star file consists position of the false positive particles. These star files contain information like X-coordinate, Y-coordinate, Angle-Psi, Origin X (Ang), Origin Y (Ang), Defocus U, Defocus V, Defocus Angle, Phase Shift, CTF B Factor, Optics Group, and Class Number of the particles.

To ensure the reproducibility of the dataset, we have included the intermediate data (Fig. 10, *ground_truth >> intermediate_data*) along with the intermediate metadata (presented in Supplementary Table S2). The intermediate data comprise the star files of manually expert-picked particles, which were utilized to construct templates for further particle selection.

Besides, there is a subdirectory called *particle_coordinates* inside *ground_truth*, which contains csv files, with same name as raw micrographs. This sub-directory comprises individual protein particle’s X-Coordinate, Y-Coordinate along with their diameter and other physico-chemical information.

## Technical Validation

To ensure that the dataset is of high quality, we applied numerous validations and statistical analyses throughout the data curation process.

### Quality of data

As noted in Fig. 4, we ensure that the dataset exclusively contains micrographs obtained using the Cryo-EM technique. Only the EMPIAR IDs with resolution better than 4 Å are chosen for creating refined protein metadata and ground truth labels of protein particles. The detailed quality control procedures are described as follows.

### Distribution of data

#### Diverse protein types

To be inclusive and ensure unbiased data generation, we selected the cryo-EM data of 34 different, diverse protein types (e.g., membrane, transport, metal binding, signalling, nuclear, viral proteins) to manually label protein particles, which can enable machine learning methods trained on them to work for many different proteins in the real-world. We selected the datasets covering different particle size, distribution density, noise level, ice and carbon areas, and particle shape as they are influential in particle picking.

#### Diverse micrographs within the same protein type

The variance in micrographs’ defocus values within a EMPIAR dataset is not accounted for by majority of the particle picking methods. This defocus variation causes the same particles to appear differently, altering the noise statistics of each micrograph. This makes it challenging to create thresholds to select high quality particles. Figure 7 shows an example how different defocus values impact the appearance and quality of Cryo-EM images in the same EMPIAR dataset. Therefore, during manually picking the particles, we included a wide variety of defocus levels and CTF fit.

**Fig. 11:**
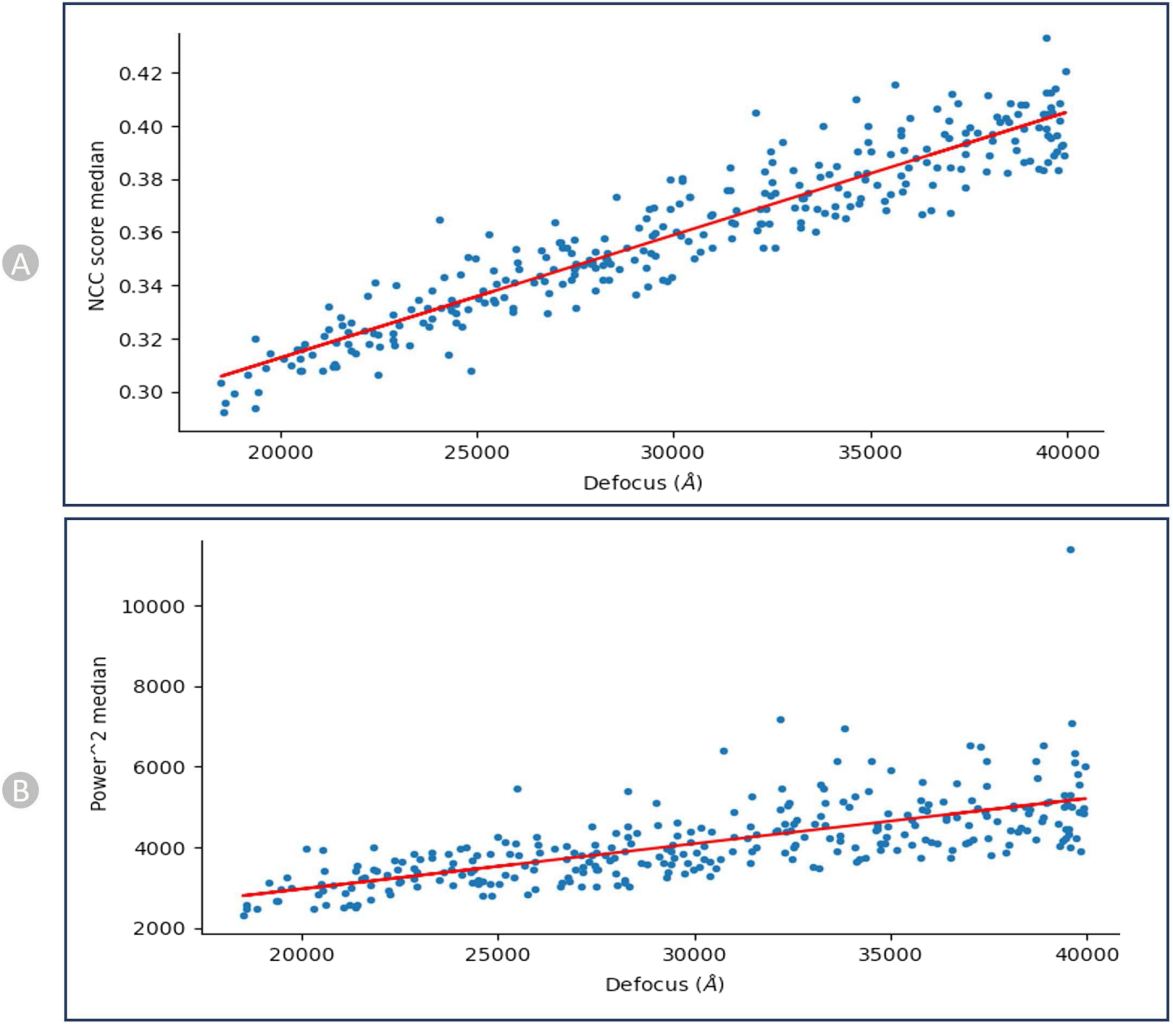
NCC and Power calibration plots for EMPIAR- 10590 (Endogenous Human BAF Complex). (**A**) Calibrating Median NCC scores vs defocus. (**B**) Calibrating Power scores vs Defocus. There is a strong trend that higher defocus correlates with higher NCC scores and same with Power score.

We recorded the correlation between defocus levels and the pick scores/the power scores (shown in Fig. 11 for EMPIAR**-**10590^51^) to assess the shape and density of a particle candidate independently. After calibration, the scores of each particle are recorded relative to the calibration line, and these values are used to define thresholds on the parameters.

### Reliability of ground truth annotations

#### Legitimacy of importing micrographs and motion correction data

All the input parameters used to prepare for loading micrographs into the CryoSPARC system were gathered from the appropriate literature. We adhered to the standards in the publications including data acquisition and imaging settings such as the microscope used, defocus range, spherical aberration, pixel spacing, acceleration voltage, electron dose and the correct usage of motion correction. Based on the microscope settings during the imaging process, we applied appropriate geometrical transformations. The defect files and the motion-correction files were flipped left-to-right or top-to-bottom and also rotated by specific degrees in clockwise/anti-clockwise direction as required. All these factors were thoroughly investigated and used during the data loading process in CryoSPARC.

#### Inspection of picked protein particles

**Fig. 12:**
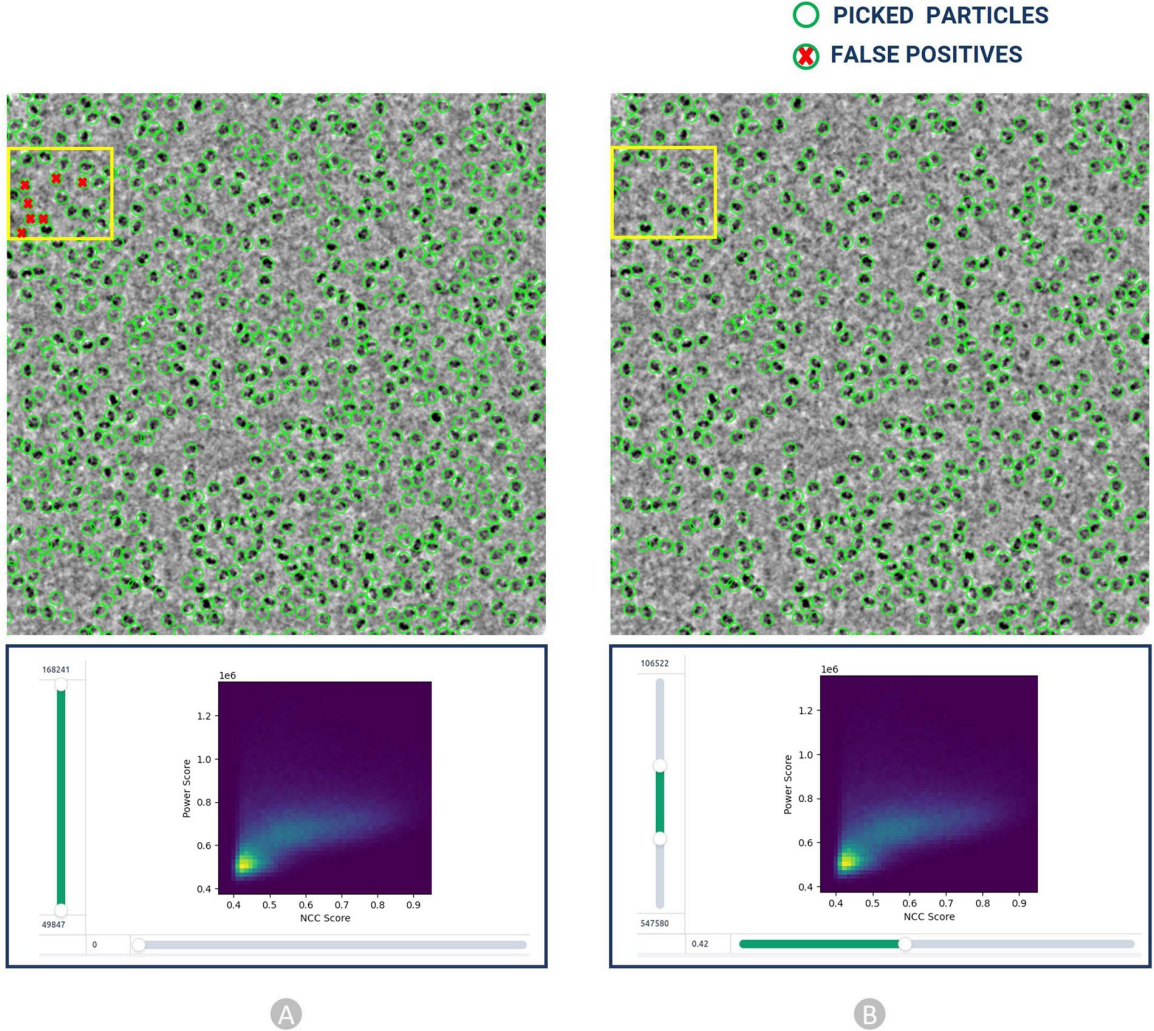
Particle inspection and filtration by adjusting normalized cross correlation (NCC) score (X axis) and local power (Y axis) for EMPIAR 10017. (**A**) Intermediate picked particles (green circles) from template-based picking step. The false positives, represented by red crossed particles inside the yellow box, are eliminated in step B. (**B**) Selected high quality true protein particles through adjustment of NCC and power score values.

The picked particles were inspected using a 2D colored histogram, as shown in Fig. 12. A particle of interest would have an intermediate local power score and a high template correlation (indicating its shape closely matches its template). Low local power scores indicate empty ice patches, even though it might resemble the template. Additionally, very high local power scores indicate carbon edges, aggregates, contaminants, and other objects with excessive densities that resemble particles.

As shown in Fig. 12 (**B,** **bottom**), we interactively specified the upper and lower thresholds for both the Power score and NCC score for each dataset improving the accuracy in the manual particle picking.

#### Cross-validation by two human experts

**Fig. 13:**
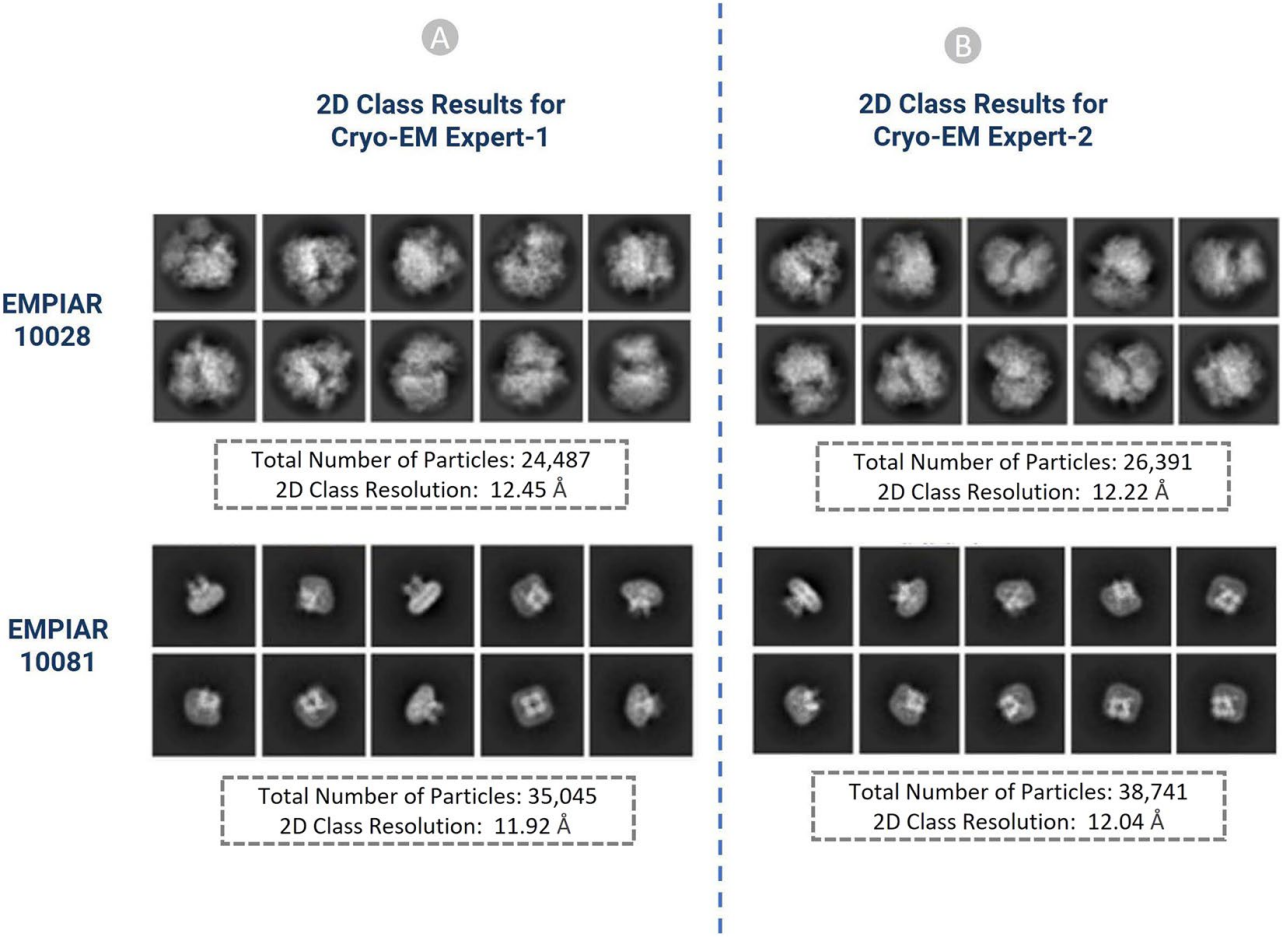
2D classification results of the picked particles of EMPIAR ID 10028 and 10081 (**A**) Results from Cryo-EM expert-1, (**B**) Results from Cryo-EM expert-2.

The results of the particles picked by the two Cryo-EM experts were compared to each other to make sure they are consistent. For example, two EMPIAR IDs: EMPIAR-10028^52^ and EMPIAR-10081^53^ with 300 micrographs (total 600 Cryo-EM micrographs) were used in cross-validation. The results of the 2D classes were compared based on total number of particles in each class, relative resolution of particles in the class, and distinct views of the structure of particles. Similar 2D classes, as shown in Fig. 13, achieved by two independent Cryo-EM specialists validate the accuracy of the manually labelled particles.

#### Comparison with existing state-of-the-art AI methods of particle picking

We conducted a comparison between the 3D density maps reconstructed from the particles picked by us and by two AI methods, namely Topaz and crYOLO. We utilized them to predict particles for two datasets, EMPIAR 10081 and EMPIAR 10345, each containing 300 micrographs. Subsequently, the ab-initio density map reconstruction and homo-refinement were performed using the generated star files containing the picked particles. To avoid any bias in the comparison, we repeated the ab-initio 3D reconstruction trials with three different random seeds for each method. The GSFSC resolution results for the three trails for each method are presented in Table 2. CryoPPP outperforms both Topaz and crYOLO.

**Table 2:** Comparison of CryoPPP, Topaz, and crYOLO on two EMPIAR DATASETS.

EMPIAR ID	Metric	Topaz			crYOLO					CryoPPP				
10081	Number of Particles Picked	135,978			17,550					37,387				
	GSFSC Resolution (Å)	Trial 1	Trial 2	Trial 3	Trial 1		Trial 2		Trial 3	Trial 1	Trial 2		Trial 3	
		6.90	**6.75**	6.96	9.56		9.61		**9.33**	**4**.**59**	4.67		4.63	
10345	Number of Particles Picked	40,324			4,095					15,894				
	GSFSC Resolution (Å)	Trial 1	Trial 2	Trial 3	Trial 1	Trial 2		Trial 3		Trial 1		Trial 2		Trial 3
		3.92	**3.85**	3.89	10.3	**9.29**		10.26		**3**.**76**		3.78		3.81

Bold font denotes the best resolution of the density map reconstructed from picked particles in the three trials.

For EMPIAR 10081, Topaz picked around 100,000 more particles than we. However, the best resolution of the density map reconstructed from CryoPPP picked particles in the three trials is 4.59 Å, substantially better than 6.75 Å of Topaz. This suggests that Topaz may have selected a substantial number of false positives (e.g., ice contaminations) or may have selected the same protein particles multiple times with slightly different center positions. CrYOLO, on the other hand, picked a significantly lower number of protein particles than us, which resulted in the worst resolution (9.33 Å) among the three. Similar results were obtained on EMPIAR 10345. The results confirm that the labeled particles in CryoPPP are of high quality and can be used to train/improve AI particle picking methods.

### Cross validation with gold standard particles picked by the authors

Gold standard particles are those particles that were originally picked by the Cryo-EM experts who generated the cryo-EM data. There are only a few EMPIAIR IDs deposited in EMPIAR that have both the micrographs and the gold standard particles. To validate the accuracy of our picked particles, we compared our results with the already-existing gold standard particles that are publicly available through the EMPIAR website. We carried out 2D and 3D validation for EMPIAR-10345^54^ and EMPIAR-10406^55^ to validate our particle labelling process as follows.

#### 2D particle class validation with gold standard

In order to get the gold standard 2D particles of the dataset, we downloaded the particle stack image files (.mrc) and.star file with the attributes of picked particles from EMPIAR. We used the particle stack and the star files to create the 2D classification results using CryoSPARC. Eventually, we compared our 2D class results with the gold standard. We performed the comparison based on the total number of classes, total number of picked particles, resolution, and visual orientation of the protein particle for each EMPIAR ID. Our results and the gold standard results exhibit strong correlations. It is worth noting that a high number of particles alone does not necessarily yield high resolution. Selecting a decent number of high-quality particles spanning a wide angular distribution is important for generating high 2D and 3D resolution.

**Fig. 14:**
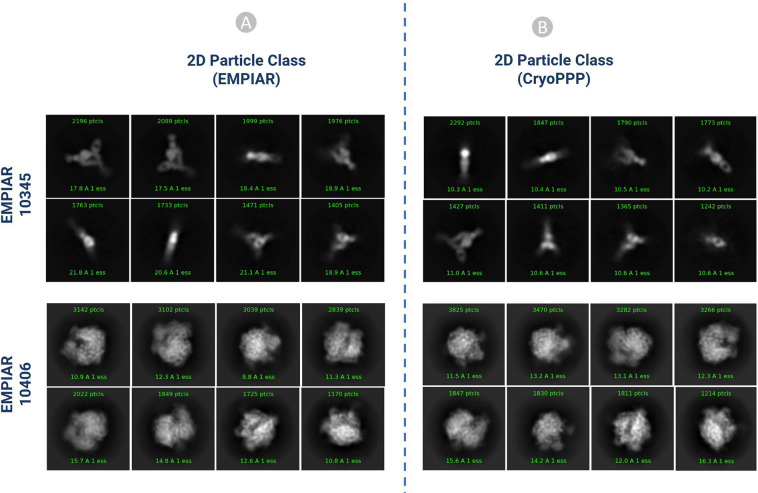
2D classification comparison for EMPIAR- 10345 and EMPIAR-10406 (**A**) 2D classification published in EMPIAR. (**B**) 2D classification results of the particles by CryoPPP.

Figure 14 shows the visual illustration 2D classification results for EMPIAR ID 10345 and EMPIAR ID 10406 published by the authors of the cryo-EM data and generated by us. They are consistent.

Table 3 compares 2D classification results generated by original authors and by us. In both cases, (Fig. 14A,B) the same 300 micrographs were used for comparison. On EMPIAR ID 10345, CryoPPP’s results have substantially better resolution than the authors’ results for both N = 50 and N = 10 classes. On EMPIAR-10406, CryoPPP’s results have better resolution for N = 50 particle classes and comparable resolution for N = 10 particle classes.

**Table 3:** 2D classification result comparison for EMPIAR-10345 and EMPIAR-10406.

EMPIAR 10345	2D Particle Class Statistics (EMPIAR)	2D Particle Class Statistics (CryoPPP)
Number of Picked Particles	17,838	15,894
Weighted Average Resolution of 2D classes (N = 50)	18.63 Å	10.25 Å
Weighted Average Resolution of 2D classes (N = 10)	20.52 Å	10.53 Å
**EMPAIR 10406**		
Number of Picked Particles	23, 450	24,703
Weighted Average Resolution of 2D classes (N = 50)	8.47 Å	7.98 Å
Weighted Average Resolution of 2D classes (N = 10)	15.53 Å	15.97 Å

#### 3D density map validation with gold standard

**Fig. 15:**
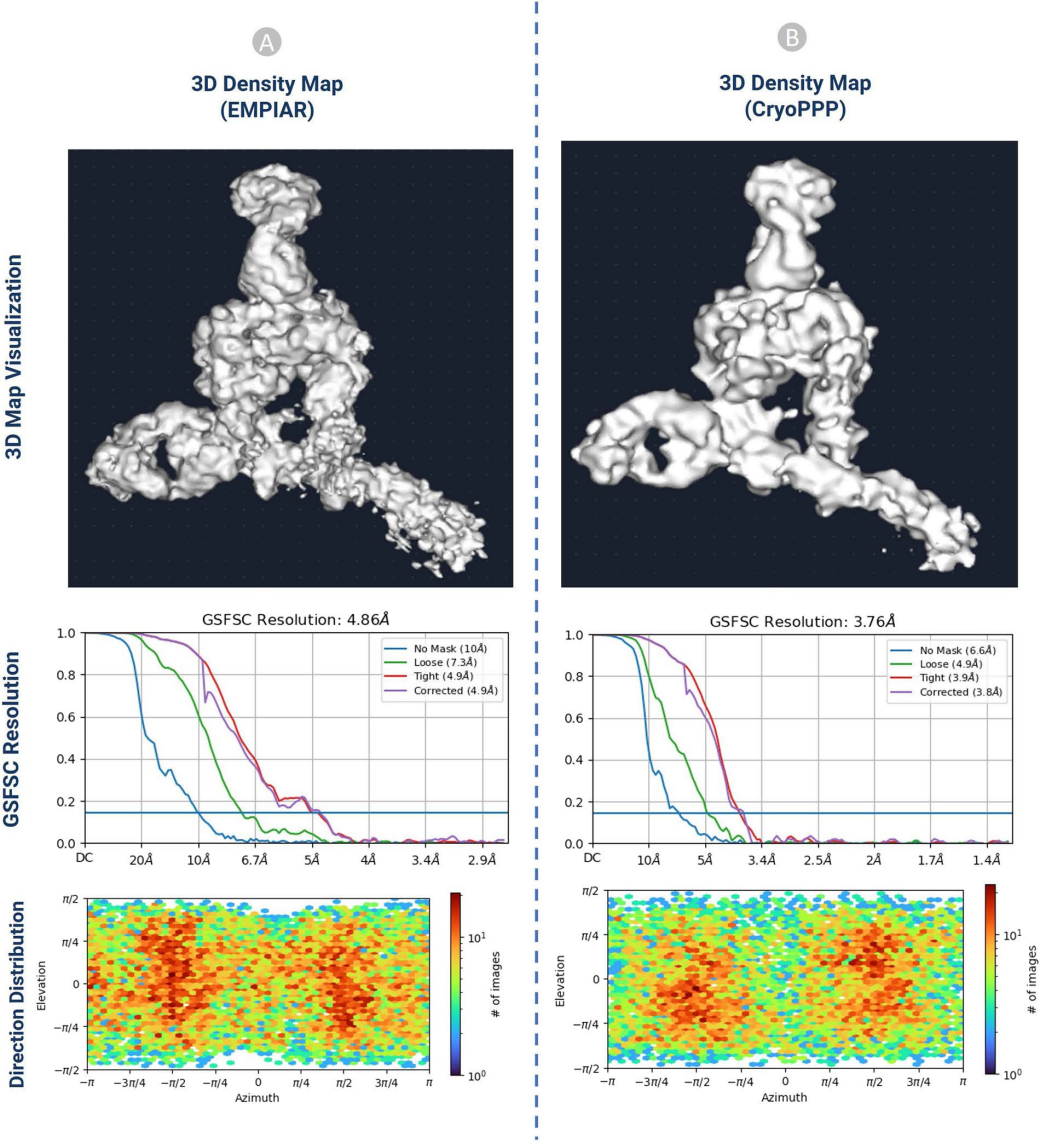
The comparison of 3D density maps, resolution, and direction distribution on EMPIAR- 10345. (**A**) results from the particles published in EMPIAR. (**B**) results generated from the particles in CryoPPP.

**Fig. 16:**
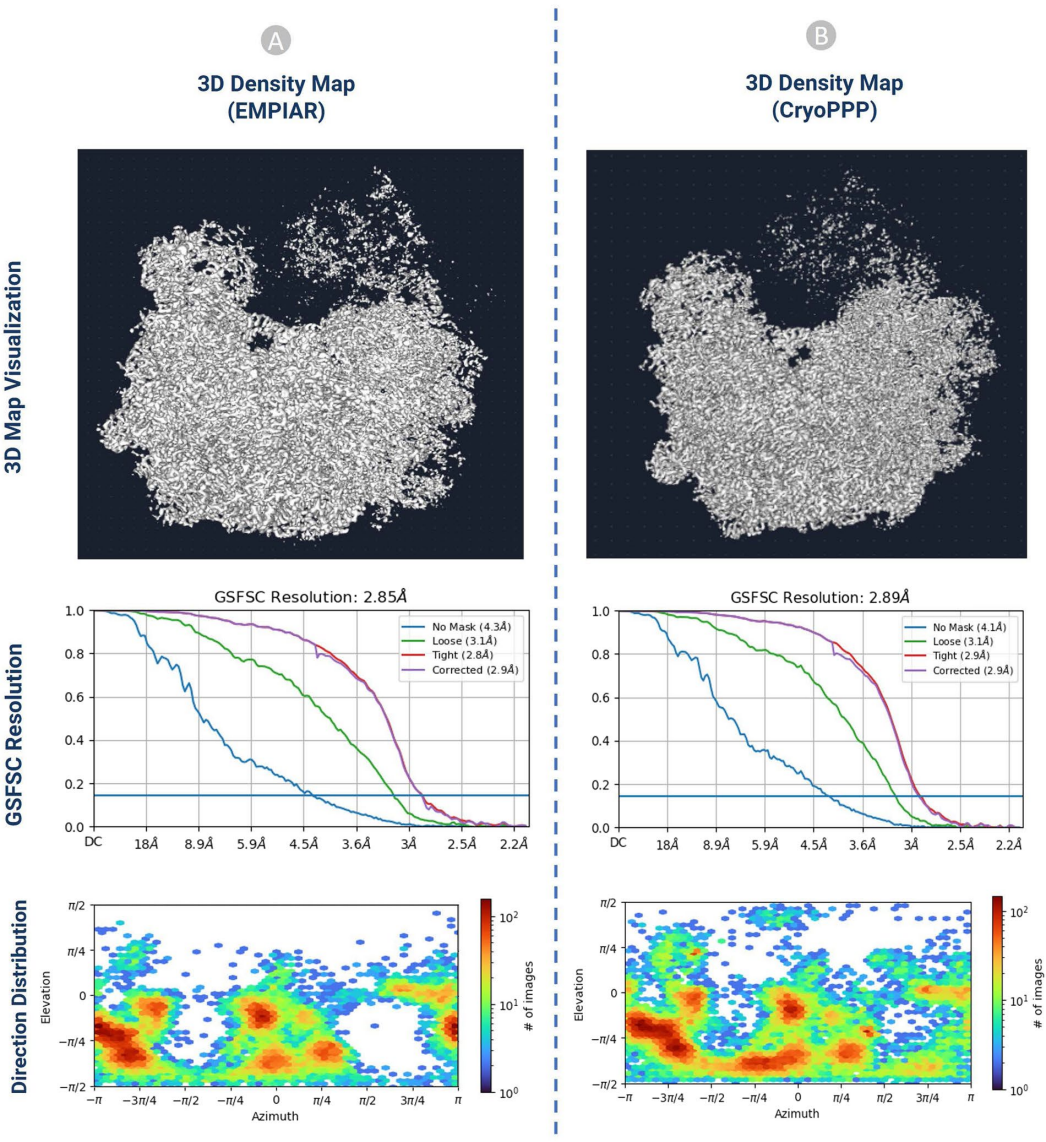
The comparison of 3D density maps, resolution, and direction distribution on EMPIAR- 10406. (**A**) results published in EMPIAR. (**B**) results generated from the particles in CryoPPP.

The ab initio reconstruction of the 3D density map for EMPIAR 10345 and EMPIAR 10406 was carried out using the gold standard particles picked by the original authors and the ones picked by us, respectively. The coordinates of the particle picked by the original authors were downloaded from the EMPIAR website. Three different random seeds were used in the ab initio reconstruction to avoid random bias. The results of the 3D density maps, resolution, and direction of distribution obtained from the two kinds of particles are compared in Figs. 15, 16.

The detailed comparison results are reported in Table 4. The ‘loose mask’ curve in the Fourier Shell Correlation (FSC) plots uses an automatically produced mask with a 15 Å falloff. The ‘tight mask’ curve employs an auto-generated mask with a falloff of 6 Å for all FSC plots.

**Table 4:** The 3D density map result comparison on EMPIAR 10345 and EMPIAR 10406.

EMPIAR 10345	3D Map Statistics (EMPIAR)			3D Map Statistics (CryoPPP)		
Number of Picked Particles	17,838			15,894		
GSFSC Resolution (Å)	Trial 1	Trial 2	Trial 3	Trial 1	Trial 2	Trial 3
	**4**.**86**	4.94	4.88	**3**.**76**	3.78	3.81
No Mask Resolution (Å)	**10**	10	10	**6**.**6**	6.5	6.6
Loose Mask Resolution (Å)	**7**.**3**	7.3	7.3	**4**.**9**	4.9	4.9
Tight Mask Resolution (Å)	**4**.**9**	4.9	4.9	**3**.**9**	3.8	3.8
Corrected Mask Resolution (Å)	**4**.**9**	4.9	4.9	**3**.**8**	3.9	3.9
**EMPAIR 10406**						
Number of Picked Particles	23, 450			24,703		
GSFSC Resolution (Å)	Trial 1	Trial 2	Trial 3	Trial 1	Trial 2	Trial 3
	**2**.**85**	2.89	2.86	**2**.**89**	2.9	2.93
No Mask Resolution (Å)	**4**.**3**	4.3	4.3	**4**.**1**	4.1	4.2
Loose Mask Resolution (Å)	**3**.**1**	3.2	3.1	**3**.**1**	3.1	3.2
Tight Mask Resolution (Å)	**2**.**8**	2.9	2.9	**2**.**9**	2.9	2.9
Corrected Mask Resolution (Å)	**2**.**9**	2.9	2.9	**2**.**9**	2.9	3.0

It is seen that CryoPPP outperforms the gold standard particles in terms of all the metrics on EMPIAR 10345 and achieves very similar results on EMPIAR 10406.

These rigorous validations with the gold standard picked particles and the comparison with the existing state-of-the-art AI methods clearly demonstrate the high quality of the data in CryoPPP, which enable it to serve as either the benchmark dataset for compare AI and classical methods of particle picking or the training and test dataset to develop new methods in the field.

## Supplementary information

Figure S1

Table S1

Table S2

Table S3

## Data Availability

The data analysis methods, software and associated parameters used in this study are described in the section of Methods. All the scripts associated with various steps of data curation are available at the GitHub repository: https://github.com/BioinfoMachineLearning/cryoppp, which includes the instructions about how to download the data.
