# Supplementary material for: A large expert-curated cryo-EM image dataset for machine learning protein particle picking
Source: Sci Data. 2023 Jun 22;10:392. doi: 10.1038/s41597-023-02280-2 (PMC10287764; doi:10.1038/s41597-023-02280-2)
Supplement: Supplementary file 1 — Figure S1 [file 41597_2023_2280_MOESM1_ESM.docx]

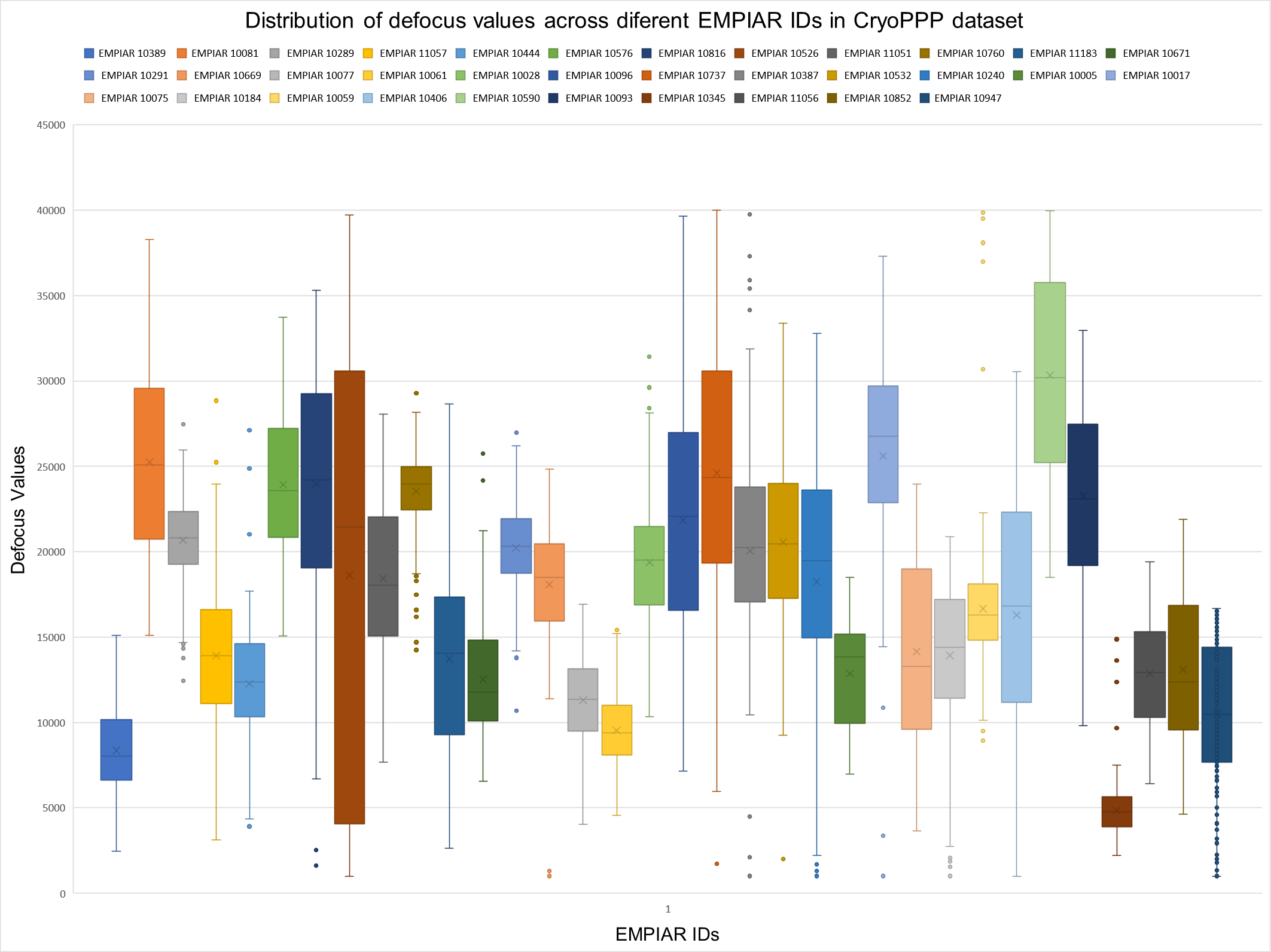


**Figure S1:** Box and whisker plot showing the distribution of Defocus Values across different EMPIAR IDs. The defocus value is represented by the central line, with the box indicating the interquartile range (IQR) and the whiskers extending to the highest and lowest values within 1.5 times the IQR. Unusual defocus values that deviate significantly from the rest of the data are displayed as individual data points beyond the whiskers.
